# Exopolysaccharides from *Lacticaseibacillus rhamnosus* Fmb14 Ameliorate Fructose-Induced Hyperuricemia and Fatty Liver via Gut Modulation

**DOI:** 10.3390/foods15030409

**Published:** 2026-01-23

**Authors:** Hongyuan Zhao, Zihan Zhang, Xiaoyu Chen, Chao Tang, Li Song, Zhaoxin Lu, Yingjian Lu

**Affiliations:** 1 School of Life Science and Engineering, Lanzhou University of Technology, Lanzhou 730050, China; 20230064@lut.edu.cn (H.Z.); a15169838673@163.com (Z.Z.); 2 College of Food Science & Technology, Nanjing Agricultural University, Nanjing 210095, China; 10410010@henu.edu.cn (X.C.); 008155@yzu.edu.cn (C.T.); fmb@njau.edu.cn (Z.L.); 3 Gansu Hualing Dairy Co., Ltd., Hezuo 747000, China; 18919859183@163.com; 4 National R & D Center for Edible Fungus Processing Technology, Henan University, Kaifeng 475004, China; 5 College of Agriculture, Henan University, Kaifeng 475004, China; 6 College of Food Science and Engineering, Yangzhou University, Yangzhou 225127, China; 7 College of Food Science & Engineering, Nanjing University of Finance and Economics, Nanjing 210023, China

**Keywords:** functional exopolysaccharides, hyperuricemia amelioration, gut homeostasis improvement, fatty liver alleviation

## Abstract

Fructose dietary intake is one of the most common risk factors for hyperuricemia, which is a critical threat to human health, and the lack of an effective biological intervention method is the main problem in preventing hyperuricemia caused by fructose intake. *Lacticaseibacillus rhamnosus* Fmb14 (*L. rhamnosus* Fmb14) has a fructose-metabolizing ability to produce extracellular polysaccharides (EPSs), and the yield of EPSs reached 0.50 and 0.42 g/L after 48 h of fermentation in liquid media of glucose-MRS and fructose-MRS. Six pure polysaccharide components were obtained after purification. A hyperuricemic mouse model was subsequently established by feeding a 60% high-fructose diet with potassium oxyazinate for 8 weeks, and the results revealed that *L. rhamnosus* Fmb14 and fructose-derived EPS (F-EPS) intervention significantly reduced the serum uric acid level of the model mice from 133.6 μmol/L to 106.7 to 111.0 μmol/L. The content of XOD in the liver decreased from 2188.1 ng/L in the model group to 1797.9 ng/L in the H-Fmb14 group and 1906.6 ng/L in the H-F-EPS group, alleviating fatty liver degeneration and improving intestinal barrier (increasing OCLN and ZO1 expression in colon). The abundances of *allobaculum*, *bacteroides*, *Lactobacilli prevotella*, and *clostridium*, the new potential biomarkers of fructose-induced hyperuricemia, were found to be modulated after Fmb14 and F-EPS intervention. The effects of Fmb14 and F-EPS in reducing uric acid synthesis and protecting the intestinal tract are very promising as food intervention agents in the prevention of hyperuricemia caused by fructose dietary.

## 1. Introduction

Hyperuricemia is one of the most serious metabolic disorders caused by multiple factors, including genetic and dietary habits [1]. In addition to genetic factors, a high-purine diet is known as the most dangerous risk factor for hyperuricemia development, but many studies have shown that a high-fructose diet is another contributor that has been neglected [2]. Fructose is a major component of many fruits and is widely used as a primary sweetener in various industrial food products to improve taste, and extra fructose intake contributes to hyperuricemia by stimulating nucleotide metabolism disorders [3]. Under normal circumstances, the fructose ingested is metabolized into glycogen and triglycerides in the liver. The excessive fructose intake leads to rapid generation of fructose-1-phosphate through ketohexokinase, which is referred to as the fructokinase C (KHK-C) or KHK enzyme. The aforementioned process brings about a decrease in ATP and phosphorylation levels, resulting in intracellular phosphate and ATP reduction in the liver [4]. This rapid reduction stimulates the increased IMP is further metabolized to inosine and hypoxanthine, both of which are the precursors of uric acid. Additionally, fructose intake damages the kidney and disrupts the uric acid transport system by obstructing excretion and increasing reabsorption [5]. In addition to industrial food production, daily fructose consumption has also increased via soft drinks and juice consumption, both of which have become increasingly popular among young adults in recent decades [4]. In addition, fructose hyperuricemia is considered more likely related to other metabolic syndromes, such as nonalcoholic fatty liver [6] and chronic renal disease [7].

Hyperuricemia can induce gout, inflammation, and other metabolic syndromes [1], but patients are only recommended for treatment after symptoms appear, and clinical therapeutic regimens often use allopurinol and probenecid, two drugs aimed at inhibiting uric acid synthesis and promoting uric acid excretion, respectively [8]. With respect to the side effects of drug treatment on the human body, new biological prevention or treatment methods, such as probiotics or bioactive compound intervention, are currently receiving increasing attention. Lactic acid bacteria have been used to promote human health for many years because of their ability to produce multiple metabolites and modulate the gut microbiota [9,10,11]. *Limosilactobacillus fermentum* JL-3 was found to degrade uric acid directly [12], *Lacticaseibacillus rhamnosus* Fmb14 was found to competitively consume purine precursors within the host [13], and *Lactobacillus brevis* DM9218 was found to manipulate intestinal dysbiosis, all of which demonstrated the potential of probiotics for ameliorating hyperuricemia [14]. Some naturally obtained carbohydrates exhibit excellent antihyperuricemic performance. A water-soluble polysaccharide composed of glucuronic acid, glucose, galactose, arabinose, and xylose obtained from *Lonicera japonica* has good antihyperuricemic ability [15]. Another novel purified polysaccharide from *Ulva lactuca* was reported to increase UA (uric acid) excretion by upregulating ABCG2 and downregulating GLUT9 [16]. The administration of polysaccharides obtained from *Enteromorpha prolifera* (EPP) could both alleviate hypoxanthine-induced hyperuricemia and regulate the dysbiosis of the gut through *Parasutterella* modulation [17].

Fructose dietary intake and the related hyperuricemia may cause disorders of gut homeostasis, including gut microbiota dysbiosis and gut barrier dysfunction. Fructose dietary intake has been reported to affect the expression of GLUT9 and URAT1 in the renal system and the gut, both of which act as transporters for excretion and reabsorption of uric acid [18,19]. Although more research has been conducted on the transport of uric acid in the kidneys, there is still evidence suggesting that the intervention of natural compounds has the potential to influence the expression of these transporters [20]. Based on the characteristics of fructose utilization [21] and exopolysaccharide secretion ability [22], lactobacilli exopolysaccharides have potential in preventing and treating fructose diet-induced hyperuricemia. The exopolysaccharides produced by *Lactobacillus plantarum* reportedly inhibited 37% of XO activity in vitro, which indicates the ability to resist gout [23]; however, the specific ability of exopolysaccharides to ameliorate diet-induced hyperuricemia in vivo has not been previously investigated. The polysaccharides may relieve the gut dysbiosis induced by hyperuricemia through gut barrier enhancement, which could regulate the expression of uric acid transporters [23]. The antihyperuricemic function of polysaccharides is commonly considered to be related to their structure, including their molecular weight and monosaccharide composition [16,17].

The previous research mostly focused on the interaction between probiotics or the exopolysaccharide with intestinal homeostasis, a lack of analysis of the direct relationship between natural active substances and uric acid metabolism and transport, especially the fructose-induced uric acid metabolism dysfunction. A probiotic with purine, fructose-metabolizing and gut-regulating abilities has been screened from traditional fermented yogurt [24]. The two characteristics of the Fmb14, including fructose utilization capacity and intestinal homeostasis regulatory ability, may enable this probiotic to play a very important role under the condition of a special excessive fructose diet in fructose-induced hyperuricemia prevention. To address the current issue of preventing the asymptomatic period of fructose diet-induced hyperuricemia and related complications through biological methods, the hyperuricemia amelioration mechanism of *L. rhamnosus* Fmb14 in a fructose dietary condition will be deeply investigated in this study.

## 2. Materials and Methods

### 2.1. Preparation of the Strain and Medium Component

*Lacticaseibacillus rhamnosus* Fmb14 (NCBI accession number: CP101845) was obtained from the Food Biotechnology Laboratory, Nanjing Agriculture University, Nanjing, Jiangsu province, China. The *L. rhamnosus* Fmb14 was activated in the MRS broth as previously described [25]. Specifically, the *L. rhamnosus* Fmb14 was streaked for purification on MRS solid medium (glucose: 20 g, beef extract: 10 g, peptone: 10 g, yeast powder: 5 g, sodium acetate: 5 g, manganese sulfate: 0.25 g, magnesium sulfate: 0.58 g, ammonium citrate dibasic: 2 g, dipotassium phosphate: 2 g, agar: 20 g, Tween 80:1 mL and water to 1000 mL, pH adjusted to 7.2) three times after being taken out from the glycerol preservation solution to obtain the monoclonal colony, and then Fmb14 was activated in MRS broth at 37 °C for 24 h. The fructose, lactose and non-sugar fermentation medium was modified by MRS broth, in which fructose and lactose were used to replace glucose in the MRS broth. All the reagents mentioned above were purchased from Aladdin Biochemical Technology Co., Ltd. (Shanghai, China).

### 2.2. Production, Isolation and Purification of EPS

The EPS were isolated from the *L. rhamnosus* Fmb14 fermentation broth of G-MRS and F-MRS (use fructose instead of glucose in the MRS), and the fresh G-MRS and F-MRS medium were inoculated (1%, *v*/*v*) with activated *L. rhamnosus* Fmb14, which was incubated at 37 °C for 24 h. The crude exopolysaccharides produced from *L. rhamnosus* Fmb14 in different modified MRS broths after incubation were based on the method described by Zhu et al. [26]. The phenol sulfuric acid method [27] was used to quantify EPS in different MRS media. Specifically, the standard curve was prepared by accurately weighing 20 mg of standard dextran into a 500 mL volumetric flask and drawing 0.4, 0.6, 0.8, 1.0, 1.2, 1.4, 1.6, and 1.8 mL and then filling up to 2.0 mL with distilled water. A total of 1.0 mL 6% phenol and 5.0 mL of concentrated sulfuric acid were added, shaken well, and left to stand for 30 min. Then, the standard curve was measured with the absorbance at 490 nm (Shimadzu Co., Kyoto, Japan). A total of 1.0 mg of EPS was dissolved in 1.0 mL of distilled water, and then 1.0 mL of 6% phenol was added, and 5.0 mL of concentrated sulfuric acid was quickly added, shaken well on a vortex mixer, and left to stand for 30 min. Then, the concentration of EPS was calculated after the absorbance at 490 nm was measured.

**Figure 1:**
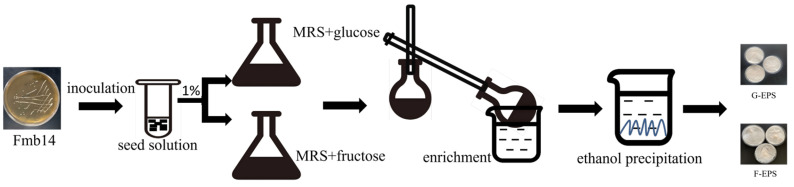
Production and isolation procedure of the extracellular polysaccharide of *L. rhamnosus* Fmb14.

Then, the G-EPS (EPS isolated from the Fmb14 fermentation broth of Glucose-MRS) and F-EPS (EPS isolated from the Fmb14 fermentation broth of Fructose-MRS) were isolated, purified, and quantified as previously described for exopolysaccharide [28,29]. Briefly, the different fermentation broths were centrifuged at 12,000× *g* (Centrifuge 5804R, Eppendorf, Hamburg, Germany) for 10 min, and then 80% (*w*/*v*) trichloroacetic acid (TCA) (Macklin Biochemical Technology Co., Ltd., Shanghai, China) was added to adjust the final supernatant as 4% (*w*/*v*) to remove the protein. The adjusted supernatant was kept at 4 °C for 6 h and then centrifuged at 12,000× *g* for 10 min to obtain the supernatant. Then, the supernatant was concentrated by rotary evaporation, and 3-fold volume of ethanol was added and maintained at 4 °C overnight to obtain a precipitate. The precipitation was re-dissolved in the deionized water and dialyzed in running water for 3 days after centrifugation at 12,000× *g* for 10 min. Finally, the crude EPS was collected after lyophilization, and the specific isolation procedure of exopolysaccharide is shown in Figure 1.

The crude EPS solutions (5 mg mL^−1^, 5 mL) were purified by a DEAE-Sepharose anion exchange column (D 2.6 cm × 50 cm) and Sephadex G-75 column (D 1.6 cm × 60 cm) (Aladdin, Shanghai, China), as previously described with some modifications [26]. Specifically, the crude EPSs were eluted with deionized water and injected into the column, the 0.1 and 0.3 M NaCl were used as eluent at a 1 mL/min flow rate, and each tube was collected every 10 min, and the content of EPSs was measured by the phenol-sulfuric method as described above. The different fractions containing purified EPS were collected for subsequent analysis after dialysis and lyophilization.

### 2.3. Characterization Analysis of EPS

#### 2.3.1. UV and FT-IR Spectra Analysis

The purified EPSs were prepared at a concentration of 1.0 mg/mL for UV spectrophotometer scanning (Shimadzu Co., Kyoto, Japan) from 190 nm to 500 nm and 1.0 mg of purified EPSs were mixed with dried KBr to get FT-IR analysis with the frequency range of 4000–500 cm^−1^.

#### 2.3.2. Monosaccharide Composition Analysis

The analysis of monosaccharide composition was performed as previously described [30]. Firstly, 5.0 mg of purified EPS was mixed with 2.0 mL trifluoroacetic acid (TFA, 2.0 M) and heated to 120 °C and kept for 2 h for hydrolysis in an oil bath. Secondly, 0.4 mL PMP solution (0.5 M) and 0.2 mL NaOH (0.3 M) were added to 0.4 mL hydrolysate, and the mixture was reacted at 70 °C for 30 min. Thirdly, 0.2 mL HCl solution (0.3 M) was added to stop the reaction after the mixture naturally cooled. The PMP derivative product was obtained in the extracted aqueous phase after adding 2.0 mL of deionized water and 4.0 mL of chloroform to the 2.0 mL of deionized water and 4.0 mL of chloroform. Finally, the monosaccharide composition of the PMP derivative product was analyzed by HPLC on a Waters 2695 system (Waters, Milford, MA, USA) with an Agilent Eclipse plus C18 column at a flow rate of 0.8 mL/min.

#### 2.3.3. Molecular Weight (Mw) Measurement [28]

The Mw and homogeneity of purified EPS fractions were determined by Agilent 1100 series HPLC with an evaporative light-scattering detector (ELSD) (Agilent 1100, Santa Clara, CA, USA). A total of 20 μL of the EPS fractions (2 mg/mL) was injected and eluted with deionized water at a flow rate of 0.8 mL/min with a TSK GEL G4000 PWXL column (column temperature 30 °C, 300 mm × 7.8 mm, Tosoh Corp., Tokyo, Japan). The standard linear curve was determined with T-series dextran standard (Aladdin, Shanghai, China).

### 2.4. The Fructose Diet Hyperuricemia Animal Model Establishment

The fructose diet-induced hyperuricemia animal model was established according to regulations controlled by the Laboratory Animal’s Research Centre of Nanjing Agriculture University, Nanjing, China. The animal experimental protocol’s approval number was NJAU. No20221011192, approved by the Ethics Committee of Nanjing Agriculture University, Nanjing, China.

The four-week-old male Kunming mice were purchased from Yangzhou University (Jiangsu, Yangzhou, China). Mice were housed normally for 7 days to allow environmental adaptation, illuminated with artificial light for 12 h every day (06:00–18:00 light cycle, 18:00–06:00 dark cycle), fed with standard laboratory food, and allowed to drink water freely. The environmental temperature was 25 °C, and the relative humidity was 60%. The environmental adaptation strategy (7 days) was carried out as previously described with some modifications [13]. A total of 64 mice were distributed into eight groups of 8 mice each after the acclimation period, and the eight groups were the control group (C), inosine-induced hyperuricemia model group (M), High/Low *L. rhamnosus* Fmb14 administration group (H-Fmb14, L-Fmb14), High/Low G-EPS administration group (H-G-EPS, L-G-EPS), and High/Low F-EPS administration group (H-F-EPS, L-F-EPS). The sample size was based on the availability of animals at the facility, and every 4 mice were fed in one cage. Activated *L. rhamnosus* Fmb14 was grown in MRS broth at 37 °C for 24 h with stationary culture, and the medium was washed with saline, and the number of viable bacteria was adjusted to 10^9^ CFU/mL and 10^7^ CFU/mL to obtain suspensions of the probiotic strains. The G-EPS and F-EPS were dissolved in saline and adjusted to 200 mg/mL.

To elicit fructose diet-induced choric hyperuricemia, the mice of the model, H/L-Fmb14, H/L-G-EPS, and H/L-F-EPS groups use 60% fructose diet (Xietong Organism, Yangzhou, China) as daily feed and orally administered with potassium oxonate (0.35 mg/100 g body weight/day) dissolved in carboxymethylcellulose sodium. Mice in H/L-Fmb14 groups were administered 100 μL *L. rhamnosus* Fmb14 dissolved in saline with concentrations at 10^9^ (H-Fmb14) and 10^7^ (H-Fmb14) CFU/mL (prepared every day). Mice in H/L-Fmb14 groups were administered G-EPS and F-EPS dissolved in saline at the dosages of 200 mg/kg/day and 100 mg/kg/day, respectively. The control group was administered the same volume of saline. The body weight of each mouse and the food and water consumption of each group were recorded.

The mice were sacrificed after 8 weeks of the experimental procedure (Figure S1A), and the sacrifice method proceeded under humane principles. Fecal and urine samples were collected, and all mice were fasted for 12 h before the sacrifice day. The blood of mice was collected from the eye vein, and the mice were sacrificed by spinal dislocation. Whole blood of mice was firstly undisturbed for 30 min and centrifuged at 3500× *g* for 20 min, and then the mice serum was collected from the supernatant. Meanwhile, the viscera sample was weighed after collection and stored at −80 °C until analysis.

### 2.5. Analysis of Biochemical Parameters in Serum and Tissues

UA and cytokines were tested in the fresh serum of mice by uric acid, IL-1β, IL-6, IL-10, NF-κB, and TNF-α ELISA kits (Jiangsu MEIMIAN Industrial Co., Ltd., Yancheng, China) according to the manufacturer’s protocol. XOD activity, BUN, glucose, GSP, TC, TG, HDL, LDL, GSH-PX, SOD, GPT, and GOT in the serum were tested by commercial kits (Jiancheng, China) according to the protocols provided. All indexes were performed on at least six samples from each group for an independent assay.

### 2.6. Histological and Immunohistochemistry Fluorescence (IF) Analysis

The intestines, colons, and liver samples were rinsed with PBS and taken with a length of 1 cm; all the collected samples were fixed with a 4% neutral formaldehyde solution and then embedded in paraffin using a tissue embedding procedure. All the samples were sent to Hycell Biotechnology (Nanjing, China). The colon, intestine, and liver were stained with hematoxylin and eosin (HE), and the liver was stained with oil red O. IF staining of colon paraffin sections was performed as previously described [24]. Five visual fields were picked randomly in three samples in each group under a microscope (NIKON ECLIPSE 80i, Nikon Co., Kyoto, Japan), and Image J software (Version 1.5) was used for semi-quantitative analysis.

### 2.7. SMRT Analysis of Gut Microbial Composition

Twenty-five colon content samples, every five samples from the control, model, H-Fmb14, H-G-EPS, and H-F-EPS groups, were collected and immediately frozen at −80 °C until DNA extraction. The procedures of DNA extraction of colon content samples were based on our previous research with some modifications [24]. The V3–V4 regions of 16S rRNA genes of the community were sequenced with forward 5′-CCTACGGGNGGCWGCAG-3′ and reverse 5′-GGACTACHVGGGTATCTAAT-3′ by the Pacbio Sequel platform at Personalbio, Inc. (Shanghai, China). All sequences were clustered into operational taxonomic units (OTUs) at a similarity level of 97%. The project number for this sequencing and analysis on Geneclouds of Personalbio (https://www.genescloud.cn/home, accessed on 15 December 2022) is MD202210202005Z8C2.

### 2.8. Statistical Analysis

The data was presented as the means ± standard derivation (SD), and statistical analysis was performed using SPSS v.17.0 software. Data analysis of the research used a one-way analysis of variance (ANOVA), and the differences between different groups were analyzed by one-way ANOVA using Tukey’s method (SPSS 16.0). The composition of the bacterial community was determined by PCoA (Rversion v.3.3.2).

## 3. Results

### 3.1. Extraction and Purification of EPS from L. rhamnosus Fmb14

**Figure 2:**
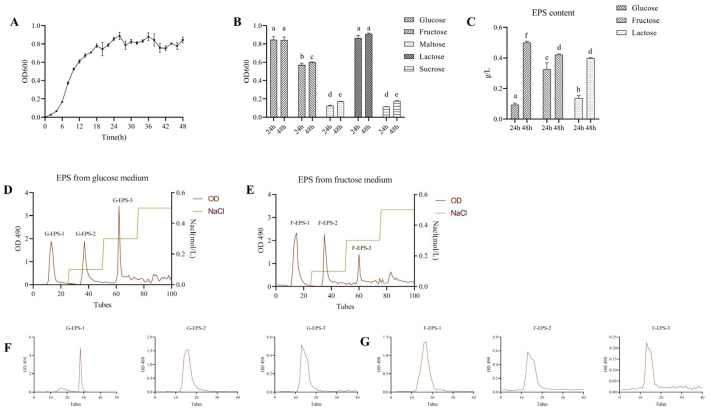
Isolation and purification of exopolysaccharides. (**A**): Growth curve of Fmb14; (**B**): Growth performance in different carbon sources; (**C**): Exopolysaccharides content in different carbon sources; (**D**,**E**): The chromatogram of DEAE-cellulose-52 anion exchange of G-EPS and F-EPS; (**F**,**G**): The chromatogram of Sephadex G-75 of G-EPSs (**F**) and F-EPS (**G**). The means ± SDs (*n* > 3 per group) are represented by bars, and different lowercase letters (abcdef) represent significant differences at *p* < 0.05; labels a to f represent lower to upper levels, respectively.

The growth curve of *L. rhamnosus* Fmb14 in MRS broth was generated, and the results revealed that *L. rhamnosus* Fmb14 was in the logarithmic growth phase from 6 to 24 h and began to plateau at 24 h (Figure 2A). To evaluate the growth ability of *L. rhamnosus* Fmb14 in different carbon sources, the OD 600 was measured at 24 and 48 h in MRS, in which glucose was replaced with fructose, maltose, lactose, and sucrose. *L. rhamnosus* Fmb14 had a good utilization rate of glucose, lactose, and glucose (*p* > 0.05), and the growth rate of *L. rhamnosus* Fmb14 in fructose-MRS broth reached 75% of that in glucose (Figure 2B), indicating that *L. rhamnosus* Fmb14 could partly use fructose to maintain growth and metabolism. The yield of crude EPS produced by *L. rhamnosus* Fmb14 reached 0.32 and 0.42 g/L after 24 and 48 h of fermentation in fructose-MRS broth through deproteinization, concentration, ethanol precipitation, and free drying (Figure 2C). EPSs were subsequently purified by using DEAE-52 cellulose and Sephadex G-75. As shown in Figure 2D–G, six single components were purified (F-EPS-1, F-EPS-2, F-EPS-3, G-EPS-1, G-EPS-2, and G-EPS-3).

### 3.2. Structure of G-EPSs and F-EPSs Produced from L. rhamnosus Fmb14

**Figure 3:**
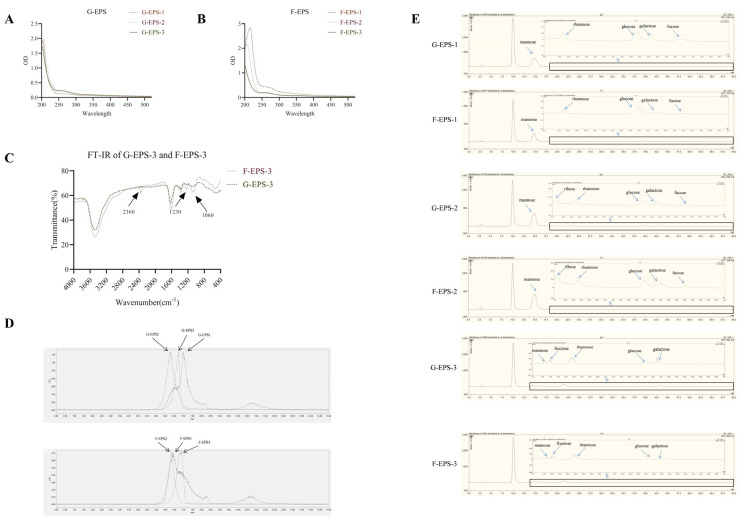
UV (**A**,**B**) and FT-IR (**C**) spectra, monosaccharide compositions (**D**), and HPLC chromatograms of the average Mw distributions (**E**) of G-EPSs and F-EPSs from *L. rhamnosus* Fmb14.

The characteristic UV spectra of G-EPS-1–1 to 3 and F-EPS-1–1 to 3 were observed from 190–500 nm (Figure 3A,B). All EPS samples exhibited no absorption peaks at 260 nm or 280 nm, indicating that the EPS samples did not contain nucleic acids or proteins. The FT-IR spectroscopy analysis results are shown in Figure S3, and all EPSs possess typical absorption peaks of polysaccharides, such as an obvious absorption peak at around 3400 cm^−1^ and 1600 cm^−1^, which are the O–H and C=O bond group stretching vibration. In addition, the EPS also possesses C–H bonds for the stretching vibrations at 2936 cm^−1^ and 1437 cm^−1^, and the absorption peak at 1000 cm^−1^ corresponds to the stretching vibrations of the polysaccharide skeletons C–O–C and C–O–H, which are probably generated by the ether bonds and hydroxyl groups of the pyranose ring in the sugar skeleton (Figure S3A,B). G-EPS1-2 has a similar spectrum with F-EPS1-2, but except for the common typical absorption bands, F-EPS-3 possesses three unique peaks at 1060 cm^−1^, 1230 cm^−1^, and 2360 cm^−1^ compared with G-EPS-3 (Figure 3C), which may provide the antihyperuricemia activity of the F-EPS. The strong absorption peaks at 1060 cm^−1^ correspond to the O–C–O group’s stretching vibration [31]. The weak absorption peaks at 2360 may be related to the CO_2_ generating COOH [32], and peaks at 1230 cm^−1^ may be attributed to unsymmetrical carbonyl stretching of the acetyl group [31].

The molecular weight (Mw) of polysaccharides is closely related to their function. The molecular weights of the six polysaccharides were determined via HPLC via calibration with standard dextrans, and the results are shown in Figure 3D. The average Mw values of G-EPS-2, G-EPS-3, F-EPS-2, and F-EPS-3 were 1.36 × 10^5^ Da, 1.57 × 10^4^ Da, 1.06 × 10^5^ Da, and 7.77 × 10^3^ Da, respectively. The peaks of G-EPS-1 and F-EPS-1 were not symmetrical or simple, which indicated that the two EPSs were not pure. The monosaccharide compositions of the EPSs were determined by HPLC after PMP derivatization (Figure 3E). On the basis of the retention time of standard sugars, G-EPS-2 and F-EPS-2 are mainly composed of mannose-ribose-rhamnose-glucose-galactose-fucose with molar ratios of 54.58:0.25:1.2:2.1:0.53:0.36 and 69.32:0.29:0.77:1.18:0.60:0.36, whereas G-EPS-3 and F-EPS-3 are mainly composed of mannose-fructose-rhamnose-glucose-galactose with molar ratios of 3.36:6.52:17.43:1.55:5.37 and 4.05:6.57:14.80:1.17:3.94. Although the composition is similar, the composition ratio of each monosaccharide component is different (Table 1).

### 3.3. Amelioration Effect of EPS on Fructose-Induced Hyperuricemia in Mice

During the 8-week feeding process (Figure S1A), the water and food consumption conditions of the mice are shown in Figure S1D,E. In terms of food consumption, the diet of the mice in the control group maintained a stable state, whereas the initial food intake in the high-fructose diet group was low and gradually increased with the progression of the experiment. The reason for this phenomenon may be that high fructose intake leads to increased energy metabolism in mice and decreased appetite. After a period of feeding, the body of a mouse adapts to the fructose diet, which is sweeter and better in taste than the normal diet, and then food consumption increases. The water consumption trend of fructose-induced hyperuricemia mice decreased (Figure S1F,G), which may increase uric acid levels to reduce urine excretion. The weights of the mice were recorded during the 8-week feeding process. The results are shown in Figure S1B,C. Before the end of the experiment, the average weight of the mice in the control group was 51.1 g, whereas that of the mice in the high-fructose diet model group decreased significantly to 40.35 g. There was no significant difference in body weight among the other groups (*p* > 0.05).

**Figure 4:**
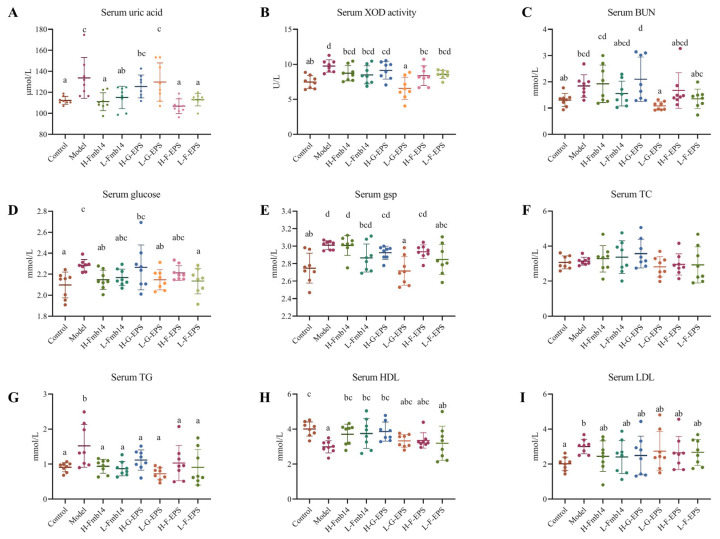
Effects of *L. rhamnosus* Fmb14 and EPS on hyperuricemic mouse serum indicators. (**A**): Serum uric acid; (**B**): Serum XOD activity; (**C**): Serum BUN; (**D**): Serum glucose; (**E**): Serum gsp; (**F**): Serum TC; (**G**): Serum TG; (**H**): Serum HDL; (**I**): Serum LDL. The means ± SDs (*n* > 6 per group) are represented by bars, and different lowercase letters (abcd) represent significant differences at *p* < 0.05; labels a to d represent lower to upper levels, respectively.

A high-fructose diet can be rapidly metabolized to form excessive ATP, which then enters the purine circulation system, resulting in an increase in the serum uric acid level. The serum uric acid concentration of the mice in the control group was 112.2 μmol/L, whereas it significantly increased to 133.6 μmol/L in the high-fructose diet group (Figure 4A). The administration of *L. rhamnosus* Fmb14 and F-EPS reduced the abnormal increase in the serum uric acid concentration induced by the fructose diet to 111.0 (H-Fmb14), 115.2 (L-Fmb14), 106.7 (H-F-EPS) and 113.0 (L-F-EPS) μmol/L. Similar results were also observed for XOD in the serum of the mice in each group. The serum XOD activity increased from 7.46 to 9.75 U/L in the control to hyperuricemic groups, and the XOD activity decreased after Fmb14 and EPS administration (Figure 4B). In addition, fructose-related biomarkers in the serum were tested, and the results revealed that the levels of BUN, glucose, glycosylated serum protein (gsp), triglyceride (TG), and low-density lipoprotein cholesterol (LDL) were lower in the serum of the model group than in that of the control group and that F-EPS decreased uric acid, XOD, BUN, glucose, TG, and LDL to control levels (Figure 4C–I).

### 3.4. Protective Effect of EPS on the Viscera of Fructose-Induced Hyperuricemic Mice

**Figure 5:**
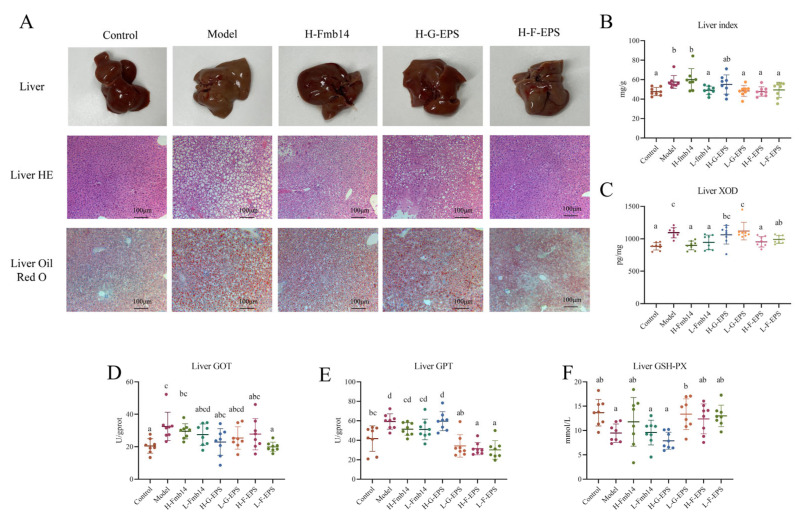
*L. rhamnosus* Fmb14 treatment reduces hyperuricemic-induced liver injury. (**A**): Apparent, H-E and Oil red O stained images of liver; (**B**): Liver index; (**C**): Liver XOD expression; (**D**): Liver GOT; (**E**): Liver GPT; (**F**): Liver GSH-PX. The means ± SDs (*n* > 6 per group) are represented by bars, and different lowercase letters (abcd) represent significant differences at *p* < 0.05; labels a to d represent lower to upper levels, respectively.

Increased serum uric acid levels are highly likely to cause kidney and liver damage. To investigate whether hyperuricemia induced by a high-fructose diet causes kidney and liver damage, H&E staining was performed on mouse kidney sections. The results revealed that fructose-induced hyperuricemia did not cause severe renal fibrosis, similar to the purine diet, and the mice in all the groups presented no lesions, and their colors were reddish brown and relatively uniform (Figure S2). Fructose diet-induced hyperuricemia led to steatosis, and H&E staining and oil red O staining further demonstrated pyroptosis and degeneration of the liver (Figure 5A,B). Compared with those of the control group, the livers of the model group showed apparent changes and became whiter, and the H-Fmb14 group showed an apparent protective effect on fatty liver. The H&E and oil red O staining results of the livers of the mice further confirmed the effect of a high-fructose diet on the development of fatty liver. The mice in the model group presented a large number of fat vacuoles, among which the H-Fmb14 and H-F-EPS interventions had significant preventive effects on fatty liver caused by a high-fructose diet, whereas the H-G-EPS intervention had a poor preventive effect (Figure 5A,B). The XOD content in the livers of the mice in the control group was 884.3 pg/mg, whereas that in the high-fructose diet group significantly increased to 1094.1 pg/mg (*p* < 0.05). The liver XOD contents of the H-Fmb14 and L-Fmb14 groups were 898.9 and 945.6 pg/mg, respectively, which were significantly lower than those of the model group (*p* < 0.05) (Figure 5C). In addition, F-EPSs also restored hepatic function by regulating GOT, GPT and GSH-PX in the liver (Figure 5D–F).

**Figure 6:**
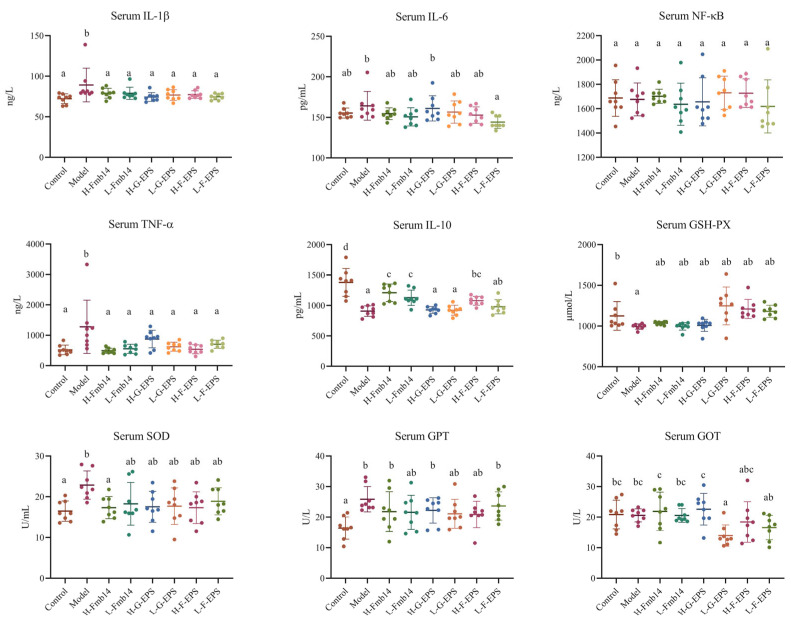
Effects of *L. rhamnosus* Fmb14 and EPS on hyperuricemic mouse serum cytokines and oxidative stress. The means ± SDs (*n* > 6 per group) are represented by bars, and different lowercase letters (abcd) represent significant differences at *p* < 0.05; labels a to d represent lower to upper levels, respectively.

Hyperuricemia may cause inflammation in the whole body of mice, thus affecting the function of various organs. The main inflammatory factors in the serum of the mice were measured, and the results are shown in Figure 6. A high-fructose diet significantly increased the serum levels of the proinflammatory factors IL-1β, TNF-α, and IL-6 and decreased the serum level of the anti-inflammatory factor IL-10 (*p* < 0.05) but had no effect on NF-κB. After the different interventions, the levels of IL-1β and IL-6 in all the intervention groups were reduced to the same level as those in the normal group, and in terms of the regulation of anti-inflammatory factors, the Fmb14 live bacteria administration group in both groups experienced significant remission (*p* < 0.05). F-EPS also ameliorated oxidative stress-induced fructose diet hyperuricemia because the abnormal levels of GSH-PX, SOD and GPT were modulated to normal levels (Figure 6).

### 3.5. Promotion Effect of EPS on Gut Barrier Dysfunction in Fructose-Induced Hyperuricemic Mice

**Figure 7:**
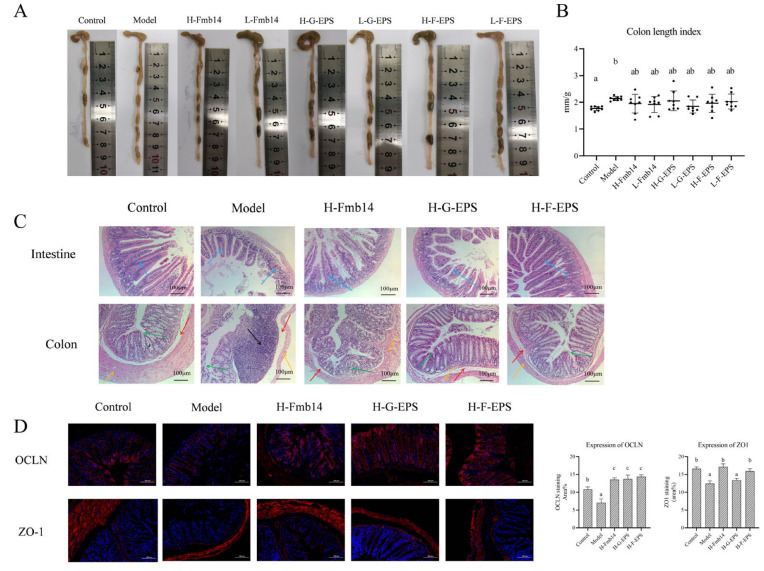
The protective effects of *L. rhamnosus* Fmb14 and EPS on gut function in hyperuricemic mice. (**A**,**B**) Images of the entire colon length of each mouse. (**C**) Hematoxylin and eosin (H-E)-stained images of the gut and colon (scale bars, 100 µm; blue arrows, villi destruction; yellow arrows, colon wall; black arrows, neutrophil infiltration; red arrows, mucosal edema; green arrows, mucosal edema). (**D**) Immunofluorescence density of the colon Zonula occludens-1 (ZO-1) and Occludin (OCLN) (bars = 50 µm, red denotes ZO-1 and OCLN, respectively). The means ± SDs and standard deviations are shown by bars (n > 5 per group), and different lowercase letters (abc) represent significant differences at *p* < 0.05; labels a to c represent lower to upper levels, respectively.

To explore the effects of hyperuricemia induced by a high-fructose diet on the gut and whether intervention with Fmb14 and exopolysaccharides has corresponding protective effects on the gut, the colon length of the mice was measured during dissection. The results are shown in Figure 7. Similar to hyperuricemia induced by a high-purine diet, a high-fructose diet also causes an increase in colon length in mice (Figure 7A,B). This may be related to the decrease in the fecal water content of the mice, while the colonic length of the mice in the *L. rhamnosus* Fmb14 group and different polysaccharide groups were restored to the control level.

To further explore the protective effects of *L. rhamnosus* Fmb14 and its EPSs G-EPS and F-EPS on the intestinal tract of mice with hyperuricemia induced by a high-fructose diet, H&E staining was performed on sections of the small intestine and colon of the mice in the high-dose treatment group, and the results are shown in Figure 5C. A large area of inflammatory infiltration appeared in the colon of the mice in the model group, while the intestinal wall became thinner, the glandular structure deteriorated, and the crypts disappeared, which led to insufficient secretion of glandular fluid in the colon, and the decrease in fecal water content made defecation difficult. At the same time, the colon became longer, and the friction between feces and the intestinal wall increased, deepening the degree of colon injury. *L. rhamnosus* Fmb14 had the most obvious protective effect on the colon. The H-Fmb14 intervention group presented a thicker colon wall, more developed glands and greater crypt depth, whereas F-H-EPS had a secondary protective effect. H&E-stained sections of the small intestine revealed that a high-fructose diet caused the small intestinal villi to shorten and the intestinal cavity to enlarge, and the decrease in intestinal absorption may be an important reason for the weight loss of the mice. In the F-H-EPS intervention group, the small intestinal structure of the mice improved, the villi became longer, and the structure was clearly visible. Intervention with *L. rhamnosus* Fmb14 live bacteria and F-H-EPS also improved the small intestine.

Many studies have shown that microbial exopolysaccharides have a positive effect on the expression of intestinal tightener proteins and that a high-fructose diet may cause destruction of the intestinal barrier, leading to intestinal absorption and excretion dysfunction, whereas *L. rhamnosus* Fmb14 has been proven to have an advantage in consolidating the intestinal barrier. The protein expression of ZO1 and OCLN in colon sections was measured via immunohistochemistry. As shown in Figure 7D, the protein level of OCLN in the colon goblet cells of the mice in the high-fructose diet group was significantly decreased (*p* < 0.05). The expression of OCLN protein in the H-Fmb14, H-G-EPS, and H-F-EPS intervention groups significantly increased and was greater than that in the control group (*p* < 0.05), which proved that *L. rhamnosus* Fmb14 and its extracellular polysaccharides play a positive role in enhancing the intestinal barrier. A high-fructose diet also decreased the protein expression of ZO1 in colon epithelial cells in the intestinal wall, but almost completely disappeared in glandular cells. The H-Fmb14 and H-F-EPS interventions increased the expression of ZO1 to the level of the control group, whereas H-G-EPS did not increase the effect of ZO1.

### 3.6. Promotion Effect of EPS on Gut Homeostasis in Fructose-Induced Hyperuricemic Mice

**Figure 8:**
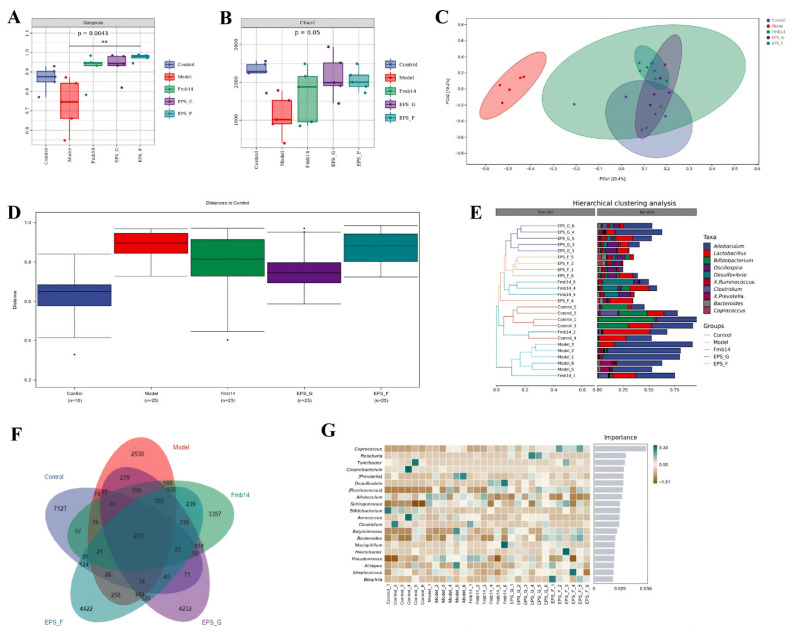
Effects of *L. rhamnosus* Fmb14 and EPS treatment on the microbial diversity of the gut microbiota in hyperuricemic mice. (**A**,**B**) Box plot of distinct groups based on the Simpson and Chao1 indices. (**C**) Principal coordinate analysis (PCoA) of the overall diversity on the basis of the Bray—Curtis distances. Scatter plot of PCoA scores depicting variances derived from bacterial communities in the five groups; (**D**) comparison of the gut microbiota between groups; (**E**) genus-level hierarchical clustering study of the gut microbiota; (**F**) Venn diagram examination of species differences in each group; (**G**) random forest species importance analysis in each group. ** represents a significant difference at the 0.99 level.

To investigate the ecological impact of fructose diet-induced hyperuricemia and evaluate the effects of *L. rhamnosus* Fmb14 and EPS administration on the diversity and abundance of the gut microbiota in mice, we obtained data from MiSeq sequencing analysis of the V3–V4 region of 16S rRNA. The α-diversity of the gut microbiota was unaltered in the model-, Fmb14-, and EPS-treated groups. The α diversity of the intestinal flora of the mice in the high-fructose diet group significantly decreased (*p* < 0.05), which was mainly manifested by decreases in the Simpson and Chao1 indices, in which a decrease in the Chao1 index represented a decrease in the flora richness of the model mice, and a decrease in the Simpson index indicated a decrease in flora diversity (Figure 8A,B). Specifically, the Simpson index of bacteria in the H-Fmb14, H-G-EPS, and H-F-EPS intervention groups was significantly greater than that in the model group and control group. Additionally, the principal coordinate analysis (PCoA) results revealed that phylogenetic community structures were markedly different between the fructose-treated and control samples and that the H-Fmb14 group included both the fructose model and control groups, indicating an obvious regulatory effect on gut microbiota dysbiosis (Figure 8C,D). Venn analysis revealed that fructose exposure significantly affected the structure of the intestinal flora and decreased the species and quantity of the gut microbiota (Figure 8F). A total of 74 shared OTUs were observed in the control and model groups; the control group had 7127 unique OTUs, whereas the model group had only 2530. Additionally, the H-Fmb14, H-G-EPS, and H-F-EPS intervention groups had 3357, 4232, and 4422 unique OUTs, respectively. The random forest analysis indicated that *Coprococcus* was the target biomarker (Figure 8F). Moreover, LfSe analysis was conducted to analyze the differences between the groups at the classification level, and the specific biomarkers within each group were obtained. Among them, *Actinobacteria* had the highest score in the LDA of the control group, while *Allobaculum*, H-Fmb14, H-G-EPS, and H-F-EPS had the highest scores in the control group in the LfSe analysis, with the highest scores in the *Ruminococcus*, *Proteobacteria*, and *Facklamia* groups, respectively (Figure 8E).

**Figure 9:**
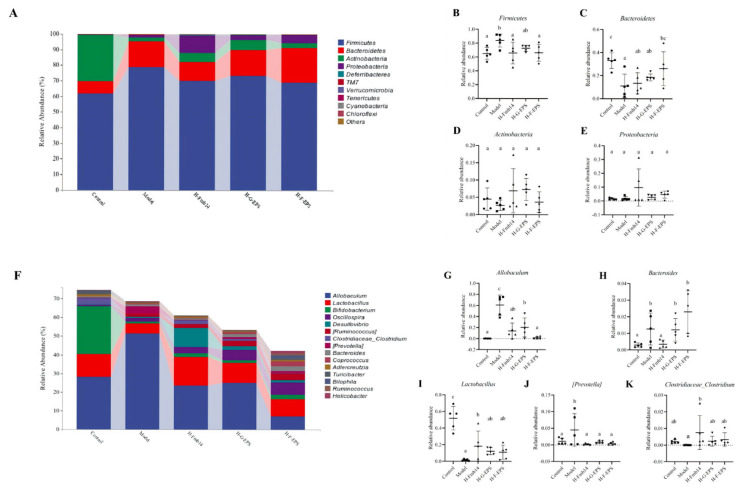
Microbial markers in *L. rhamnosus* Fmb14 and EPS prevention. (**A**): Gut microbiota phylum-level relative abundance; (**B**–**E**): Relative abundance of *Firmicutes*, *Bacteroidetes*, *Actinobacteria*, *Proteobacteria*; (**F**): Gut microbiota genus-level relative abundance; (**G**–**K**): Relative abundance of *Allobaculum*, *Bacteroides*, *Lactobacillus*, *[Prevotella]*, *Clostridiaceae_Clostridium*. The means ± SDs (*n* > 5 per group) are represented by bars, and different lowercase letters (abc) represent significant differences at *p* < 0.05.

To investigate the specific effects of Fmb14, H-G-EPS, and H-F-EPS administration on the gut microbiome under a high-fructose diet, the composition of the gut microbiome at the genus and phylum levels was analyzed, as shown in Figure 9. At the phylum level, the relative abundance of Firmicutes in the model group was significantly greater than that in the control group, increasing from 0.649 to 0.838 (*p* < 0.05). Compared with those in the normal control group, the relative abundance of Firmicutes in the Fmb14, H-G-EPS, and H-F-EPS intervention groups was restored to 0.657, 0.726, and 0.662, respectively. Additionally, the high-fructose diet caused a significant decrease in the relative abundance of Bacteroidetes in the gut microbiome of the mice, from 0.330 to 0.110 (*p* < 0.05). The relative abundance of Bacteroidetes in the H-F-EPS intervention group increased to 0.261, which was significantly greater than that in the model group (*p* < 0.05), whereas the Fmb14 and H-G-EPS interventions had no regulatory effect on the disruption of this microbial community.

## 4. Discussion

### 4.1. The Unique Characteristic of F-EPS Gives Fmb14 Potential to Alleviate Hyperuricemia

Exopolysaccharides (EPSs) are a special secondary metabolite that is produced by specific strains and has important significance for microorganisms. Lactobacilli have been reported to possess the ability to produce many bioactive EPS, and the type of carbon source is one of the most important factors affecting EPS synthesis [33]. The gene cluster on the strain determines EPS biosynthesis, and the carbon source regulates the expression of polymerase, invertase and glycosyltransferase in variable regions of the gene cluster, which may be the drivers of EPS diversity [34]. The Fmb14 produced G-EPS in the carbon source, and the two EPSs showed different biological activities. The nutritional and prebiotic properties of a food depend on its structural characteristics, and the biological activity of a food exerts different effects under different conditions [35]. Fructose is a favorable dietary component because of its high sweetness, and it can be absorbed and metabolized rapidly to provide energy for the body, which can relieve symptoms of hypoglycemia immediately [36]. However, a long-term fructose diet results in a heavy burden on the body, and obesity and fatty liver are the most common fructose-related metabolic diseases. In recent decades, increasing evidence has shown that dietary fructose is closely related to hyperuricemia [3]. Probiotics have been shown to alleviate hyperuricemia caused by a purine diet, and a probiotic supplementation strategy also effectively improves NAFLD caused by fructose. Probiotics alleviate NAFLD through immune system enhancement, intestinal flora modulation, inflammatory regulation, insulin resistance reduction, and intestinal permeability protection [37]. As a potential dietary supplement for fructose-induced hyperuricemia, the administration of *L. rhamnosus* could utilize fructose before it is absorbed in the intestine, although this effect was not the dominant factor of *L. rhamnosus* in reducing the increase in the serum uric acid level, but it still has a synergistic effect to reduce the absorbed rate of fructose. The competitive utilization of *L. rhamnosus* Fmb14 by the intestine of the host decreases XOD synthesis and ultimately reduces uric acid production.

Over the past few years, natural polymers, especially EPSs synthesized by probiotics, have received much attention from the scientific community for their numerous benefits to the gut [38]. EPSs are recognized as superior health promoters that have the potential to serve as substitutes for synthetic drugs because of their antioxidant, anti-inflammatory, and immune activation abilities and their ability to regulate intestinal homeostasis in the host [39]. The types and contents of metabolites are affected by environmental factors. *Lactobacillus plantarum* LPBF35, a fructophilic *Lactobacillus*, has been reported to contain high proportions of fructose during cacao bean fermentation, increasing the accumulation of some aroma components, such as ethyl acetate and isoamyl acetate [40]. Different structural exopolysaccharides are produced by *L. plantarum* under different carbon sources because of the different carbohydrate utilization patterns [41]. *L. rhamnosus* Fmb14 showed similar characteristics in the fermentation process in vitro as described above. The EPS yield reached 0.42 g/L after 48 h of fermentation in MRS-fructose medium, which was 82% of the total EPS yield in glucose fermentation medium by *L.rhamnosus* Fmb14. The biological activity of polysaccharides is determined by their structure, and their molecular weight is one of the key factors. Two polysaccharides named PSPA and PSPB extract from *Polygonati rhizoma* both reduce serum acid level in mice and PSPB, which has bigger molecular weight (112.2 kDa) and exhibit better ability on serum uric acid and XOD activity ability [42]. However, we obtained an opposite result, that crude F-EPS showed a superior ability to ameliorate hyperuricemia, and the minor molecular weight may have more advantages in reducing uric acid, based on our research. Although different molecular weights may provide a variety of biological activities of EPS [43], molecular weight was not the only factor that affected EPS biological activity. The FT-IR results showed that three specific peaks of bonds in F-EPS-3, which may provide the unique anti-HUA ability of F-EPS at the mouse level. Ribose and fructose emerge in the composition, both of which were not common in the EPS fractions, especially in the microbial fermented production [44]. Although the ribose usually takes a low proportion in the EPS composition, it still exhibits anti-inflammatory or immune-strengthening activity in *L. plantarum* and *S. thermophile* [45,46]. The fructose exhibited a higher proportion than ribose in the EPS according to our results, and fructose-containing EPS showed more activity, such as antioxidant, anticancer, and gut microbiota regulation [47,48,49]. The two uncommon components, especially the fructose, may provide the special ability to anti-HUA. Both low- and high-dose levan (fructosan) interventions decrease serum uric acid levels significantly through gut microbiota and serum metabolites modulation [50]. Additionally, the highly anti-HUA EPS PSP that was mentioned above was primarily composed of fructose (72.74%) by high-performance anion-exchange chromatography (HPAEC) analysis, which may reveal that fructose play an important role in EPS composition of anti-HUA activity [42]. *Bifidobacterium animalis* RH can produce extracellular polysaccharides with a molecular weight of 2.31 × 10^4^ Da, which have been found to have strong antioxidant activity in vitro, whereas polysaccharides with larger molecular weights are considered to have better antitumor activity [51]. This conclusion was consistent with a study on the antitumor activity of *Ganoderma lucidum* polysaccharides [52]. In addition, EPS secreted from *L. plantarum* 1.0665, with a molecular weight of 9.52 × 10^4^ Da, was found to have anti-gout bioactivity in vitro [23]. Three polysaccharide components were isolated from F-EPS produced by *L. rhamnosus* Fmb14, and their molecular weights were 7.77 × 10^3^ Da, 7.21 × 10^4^ Da, and 1.06 × 10^5^ Da. Although the compositions of G-EPS and F-EPS are similar, the molar ratios are quite different, and G-EPS-1, G-EPS-2, F-EPS-1, and F-EPS-2 are mainly composed of mannose, whereas G-EPS-3 and F-EPS-3 are mainly composed of rhamnose. The different molecular weights and structures of G-EPSs and F-EPSs may cause different amelioration effects on hyperuricemia in mice. The monosaccharide composition of EPS is mainly determined by the strain type and culture conditions [53], and the EPS produced by lactobacilli is mostly heteropolysaccharides, which are mainly composed of mannose and mostly contain glucose, galactose, fructose, and rhamnose [54,55]. *L. rhamnosus* has a superior ability to produce exopolysaccharides, which could improve the adhesion of mucin under different gastrointestinal conditions [56]. F-EPS administration also reduced the degree of fatty liver degeneration in high-fructose -induced mice, which was consistent with the findings of *Lactobacillus rhamnosus* ZFM231, which can be fermented to produce polysaccharides of four different components, decrease lipase activity and inhibit fat synthesis in vitro [55].

### 4.2. F-EPS Alleviated Fructose-Induced Hyperuricemia Through Multiple Pathways

Previous studies have reported that a high-fructose diet promotes uric acid production during metabolism [14,57,58], and persistent high fructose intake is the second risk factor for hyperuricemia. A stable mouse model of hyperuricemia was established by feeding a 60% fructose diet combined with potassium oxyazinate for 8 weeks, and the level of serum uric acid in the model group increased by 19.1% compared with that in the control group, whereas it was significantly reduced in the *L. rhamnosus* Fmb14- and F-EPS-treated groups (Figure 4A). Elevated serum uric acid often accompanies renal disorders [58], while a high-fructose diet is one of the risk factors for liver damage [59]. Fructose intake induces ATP consumption to produce AMP, and the accumulation of AMP stimulates the synthesis of AMP deaminase, leading to the degradation of purine nucleotides into uric acid [60,61]. This process also leads to increased expression of XOD, a key enzyme in uric acid metabolism, which is consistent with our conclusions (Figure 4B and Figure 5C). Research on bioactive compounds from marine foods for hyperuricemia prevention found that the anti-HUA biomarker of marine-related polysaccharides inhibited ADA and XOD activities or inhibited GLUT9 and URAT1 expression, and both ways were influenced by polysaccharide structures [62]. The chemical structures develop biological activities, but their relationship is complex. The NMR analysis of high anti-HUA polysaccharides PSPB from *Polygonati rhizoma* proved the existence of α- and β-anomeric protons, but it still cannot explain which provides the special biological activities. The biological activities, especially the anti-HUA activities of polysaccharides, were affected by many factors, including homogeneity, molecular weight, monosaccharide composition, functional bond and C, H-NMR spectrum [42]. To clarify the deep relationship between structure and biological activity, some new methods, like fluorescent, isotope labeling, or molecular docking at the animal level, may be meaningful. The degree of damage to the liver is greater than that to the kidney in hyperuricemia caused by high fructose because the increase in uric acid levels caused by fructose is an indirect process, and the process mainly occurs in the liver [63]. The rate of uric acid production after the intake of a large amount of fructose was lower than that after the consumption of a purine-enriched diet; thus, the rate of renal disease in fructose hyperuricemia mice decreased because more regulatory time was given to the kidney during the excretion process. Renal excretion of uric acid is regulated mainly by uric acid reabsorption proteins (GLUT9 and URAT1) and secretory proteins (ABCG2 and MRP4) [19,64], and the lower rate of renal disease allows the kidney to retain more ability to maintain uric acid excretion so that the serum uric acid level of mice fed a high-fructose diet for 8 weeks is lower than that of mice fed a high-purine diet for 8 weeks. The *Plantaginis Semen* polysaccharide (PSP) was found reduce serum uric acid and alleviate renal dysfunction through modulation of xanthine oxidase (XOD) and gut microbiota, which is the same as F-EPS works [65].

### 4.3. F-EPS Intervention Reduces Fatty Liver and Related Inflammation by Gut Modulation

Fructose has been shown to cause the accumulation of liver lipids even in a single excessive intake [66], and a long-term fructose diet can stimulate the activation of adipogenesis signaling pathways, leading to nonalcoholic fatty liver disease [67] or obesity [68]. Fatty liver and elevated uric acid induced by a high-fructose diet lead to inflammation in mice [69], while intervention with H-Fmb14 and H-F-EPS reduces the contents of IL-1β, IL-6, and TNF-α and increases the level of the anti-inflammatory factor IL-10 in the serum by alleviating fatty liver degeneration and reducing the serum uric acid level. These findings are similar to those of a previous study on the use of *Sporisorium reiliana* polysaccharides and *Phoenix dactylifera* (jujube) monosaccharides to alleviate hyperuricemia caused by a high-fructose diet [70]. In this study, dietary intervention with polysaccharides not only downregulated the transcription of the proinflammatory cytokines IL-1β and IL-6 but also inhibited the activation of the NLRP3 inflammasome complex, blocked the TLR4/MyD88/NF-κB signaling pathway, and alleviated the inflammatory response in the kidneys and livers of mice [70]. In addition to the liver and kidneys, the intestine, as the organ of fructose absorption and uric acid excretion, also plays a crucial role in hyperuricemia caused by high fructose. Although there are no reports that fructose directly causes intestinal inflammation, a study on high-fructose corn syrup revealed that a fructose diet can aggravate the intestinal inflammatory response by activating the NF-κB signaling pathway through the production of ROS [71]. A high-fructose diet induces an increase in uric acid in the blood, which stimulates low-level intestinal inflammation. Therefore, a sustained high-fructose diet exacerbates the intestinal inflammatory response.

Dysregulation of the intestinal flora may affect uric acid metabolism and lead to elevated serum uric acid levels; thus, controlling the intestinal flora through the diet is a potential target for alleviating hyperuricemia [3]. With the popularization of new sequencing methods, the relationships between various metabolic diseases and the intestinal flora have gradually been revealed. The richness and diversity of the intestinal flora in patients with gout and hyperuricemia are significantly lower than those in healthy people [72], which is consistent with the results of this study. At the phylum level, the relative abundance of Firmicutes increased while that of Bacteroidetes decreased in high-fructose diet-fed mice, but some studies reported contrary results because although all the models were induced by fructose, solid feed with 60% fructose was used in this study, whereas 13% fructose solution was used in the literature for gavage [71,73]. A high-fructose diet could have a strong effect on the intestinal flora, and the results of this study revealed a significant increase in the abundance of *Allobaculum* and a significant decrease in the abundance of lactobacilli and *Clostridiaceae_Clostridium.* Interference with Fmb14 and its exopolysaccharide significantly increased the abundance of lactobacilli, which have been reported to regulate purine metabolism and reduce the serum uric acid level [73]. Another metagenomics study on the intestinal flora of gout patients revealed an abnormal increase in the abundance of *Bacteroides* and *Prevotella* [74], which is consistent with the results of the present study, that the disturbance of the flora caused by a fructose diet is a risk factor for hyperuricemia. Intervention with *L. rhamnosus* Fmb14 significantly reduced the abundance of *Bacteroides* and *Prevotella*, but intervention with these two polysaccharides affected only the abundance of *Prevotella*. *Allobaculum* is an often overlooked bacterium in the gut microbiota, but existing studies have revealed a strong correlation with the development of obesity and fatty liver [75], especially in individuals with obesity induced by a sucrose diet [76]. According to our results, the *Allobaculum* may play more important roles in the intestinal microbiota in individuals with food source metabolic diseases, such as fructose-induced hyperuricemia.

## 5. Conclusions and Future Perspective

In summary, the effects and mechanism of *L. rhamnosus* Fmb14 and the polysaccharide F-EPS in alleviating hyperuricemia induced by a fructose diet were investigated in this study. *L*. *rhamnosus* Fmb14 plays a role in alleviating hyperuricemia mainly through competition for fructose precursors, regulation of uric acid metabolism, optimization of the intestinal flora and strengthening of the intestinal barrier. F-EPS, as a prebiotic, alleviates hyperuricemia mainly by regulating the intestinal flora and consolidating the intestinal barrier.

Using probiotics or other natural active ingredients to alleviate hyperuricemia caused by purine or fructose diets is an emerging trend. This article provides high-quality potential strains *L. rhamnosus* Fmb14 and demonstrates its excellent anti-uric acid biological activity, but there still exist some limits in the application of *L. rhamnosus* Fmb14. Our next research will focus on how to transform Fmb14 into a food additive and determine its actual effect on uric acid reduction in high-purine or fructose diets (such as seafood or high-fructose beverages) in real-life scenarios.

## Figures and Tables

**Table 1:** The Mw and monosaccharide compositions of purified EPS fractions.

Samples	Monosaccharide Compositions (%)						
	Man	Rib	Rha	Glu	Gal	Fuc	Fru
G-EPS-2	92.5	0.4	2.0	3.6	0.9	0.6	-
F-EPS-2	95.6	0.4	1.1	1.6	0.8	0.5	-
G-EPS-3	9.8	-	51.0	4.5	15.7	-	19.0
F-EPS-3	13.3	-	48.5	3.8	12.9	-	21.5

Man: mannose; Rib: ribose; Rha: rhamnose; Glu: glucose; Gal: galactose; Fuc: fucose; Fru: fructose.

## Data Availability

The original contributions presented in this study are included in the article/Supplementary Material. Further inquiries can be directed to the corresponding author.

## Supplementary Materials

The following supporting information can be downloaded at: https://www.mdpi.com/article/10.3390/foods15030409/s1.

## References

[1] Zhao H.Y., Lu Z.X., Lu Y.J. (2022). The potential of probiotics in the amelioration of hyperuricemia. Food Funct..

[2] Nakagawa T., Lanaspa M.A., Johnson R.J. (2019). The effects of fruit consumption in patients with hyperuricaemia or gout. Rheumatology.

[3] Zhang C., Li L., Zhang Y., Zeng C. (2020). Recent advances in fructose intake and risk of hyperuricemia. Biomed. Pharmacother..

[4] Taskinen M.-R., Packard C.J., Boren J. (2019). Dietary Fructose and the Metabolic Syndrome. Nutrients.

[5] Andres-Hernando A., Orlicky D.J., Kuwabara M., Ishimoto T., Nakagawa T., Johnson R.J., Lanaspa M.A. (2020). Deletion of Fructokinase in the Liver or in the Intestine Reveals Differential Effects on Sugar-Induced Metabolic Dysfunction. Cell Metab..

[6] Younossi Z.M. (2019). Non-alcoholic fatty liver disease—A global public health perspective. J. Hepatol..

[7] Sayehmiri K., Ahmadi I., Anvari E. (2020). Fructose Feeding and Hyperuricemia: A Systematic Review and Meta-Analysis. Clin. Nutr. Res..

[8] Terkeltaub R. (2010). Update on gout: New therapeutic strategies and options. Nat. Rev. Rheumatol..

[9] Plaza-Diaz J., Javier Ruiz-Ojeda F., Gil-Campos M., Gil A. (2019). Mechanisms of Action of Probiotics. Adv. Nutr..

[10] Azzahra R., Arifin H., Juwita D.A. (2024). The effect of ethyl acetate fraction of Vernonia amygdalina Del. leaves on uric acid levels of male white mice (Mus musculus) hyperuricemia. Prospect. Pharm. Sci..

[11] Birinci Yildirim A., Cimen A., Baba Y., Turker A. (2024). Natural- and in vitro-grown *Filipendula ulmaria* (L.) Maxim: Evaluation of pharmaceutical potential (antibacterial, antioxidant and toxicity) and phenolic profiles. Prospect. Pharm. Sci..

[12] Wu Y., Ye Z., Feng P., Li R., Chen X., Tian X., Han R., Kakade A., Liu P., Li X. (2021). *Limosilactobacillus fermentum* JL-3 isolated from “Jiangshui” ameliorates hyperuricemia by degrading uric acid. Gut Microbes.

[13] Zhao H., Chen X., Meng F., Zhou L., Pang X., Lu Z., Lu Y. (2023). Ameliorative effect of *Lacticaseibacillus rhamnosus* Fmb14 from Chinese yogurt on hyperuricemia. Food Sci. Hum. Wellness.

[14] Wang H.N., Mei L., Deng Y., Liu Y.H., Wei X.Q., Liu M., Zhou J.R., Ma H., Zheng P.Y., Yuan J.L. (2019). *Lactobacillus brevis* DM9218 ameliorates fructose-induced hyperuricemia through inosine degradation and manipulation of intestinal dysbiosis. Nutrition.

[15] Yang Q., Wang Q., Deng W., Sun C., Wei Q., Adu-Frimpong M., Shi J., Yu J., Xu X. (2019). Anti-hyperuricemic and anti-gouty arthritis activities of polysaccharide purified from *Lonicera japonica* in model rats. Int. J. Biol. Macromol..

[16] Li X., Chen Y., Gao X., Wu Y., El-Seedi H.R., Cao Y., Zhao C. (2021). Antihyperuricemic Effect of Green Alga *Ulva lactuca* Ulvan through Regulating Urate Transporters. J. Agric. Food Chem..

[17] Li X., Gao X., Zhang H., Liu Y., Sarker M.M.R., Wu Y., Chen X., Zhao C. (2021). The anti-hyperuricemic effects of green alga *Enteromorpha prolifera* polysaccharide via regulation of the uric acid transporters in vivo. Food Chem. Toxicol..

[18] Chen Y., Liu Q.P., Meng X.Y., Zhao L.Q., Zheng X.K., Feng W.S. (2024). Catalpol ameliorates fructose-induced renal inflammation by inhibiting TLR4/MyD88 signaling and uric acid reabsorption. Eur. J. Pharmacol..

[19] Novikov A., Fu Y., Huang W., Freeman B., Patel R., van Ginkel C., Koepsell H., Busslinger M., Onishi A., Nespoux J. (2019). SGLT2 inhibition and renal urate excretion: Role of luminal glucose, GLUT9, and URAT1. Am. J. Physiol.-Ren. Physiol..

[20] Jia L.Y., Sun B.Y., Nie A.Z., Shi Y.M., Zhou Z., Zhu C.S. (2025). Rosmarinic acid attenuates hyperuricemia via restoring hyperuricemia-induced renal and intestinal dysfunctions. Phytomedicine.

[21] Oh J.-H., Alexander L.M., Pan M., Schueler K.L., Keller M.P., Attie A.D., Walter J., van Pijkeren J.-P. (2019). Dietary Fructose and Microbiota-Derived Short-Chain Fatty Acids Promote Bacteriophage Production in the Gut Symbiont Lactobacillus reuteri. Cell Host Microbe.

[22] Saadat Y.R., Khosroushahi A.Y., Gargari B.P. (2019). A comprehensive review of anticancer, immunomodulatory and health beneficial effects of the lactic acid bacteria exopolysaccharides. Carbohydr. Polym..

[23] Jiang B., Wang L., Zhu M., Wu S., Wang X., Li D., Liu C., Feng Z., Tian B. (2021). Separation, structural characteristics and biological activity of lactic acid bacteria exopolysaccharides separated by aqueous two-phase system. Lwt-Food Sci. Technol..

[24] Zhao H., Chen X., Zhang L., Meng F., Zhou L., Pang X., Lu Z., Lu Y. (2022). *Lacticaseibacillus rhamnosus* Fmb14 prevents purine induced hyperuricemia and alleviate renal fibrosis through gut-kidney axis. Pharmacol. Res..

[25] Zhao H., Chen X., Zhang L., Tang C., Meng F., Zhou L., Zhu P., Lu Z., Lu Y. (2023). Ingestion of *Lacticaseibacillus rhamnosus* Fmb14 prevents depression-like behavior and brain neural activity via the microbiota-gut-brain axis in colitis mice. Food Funct..

[26] Zhu Y., Wang C., Jia S., Wang B., Zhou K., Chen S., Yang Y., Liu S. (2018). Purification, characterization and antioxidant activity of the exopolysaccharide from *Weissella cibaria* SJ14 isolated from Sichuan paocai. Int. J. Biol. Macromol..

[27] Kimmel S.A., Roberts R.F., Ziegler G.R. (1998). Optimization of exopolysaccharide production by *Lactobacillus delbrueckii* subsp. bulgaricus RR grown in a semidefined medium. Appl. Environ. Microbiol..

[28] Tian J., Zhang C., Wang X., Rui X., Zhang Q., Chen X., Dong M., Li W. (2021). Structural characterization and immunomodulatory activity of intracellular polysaccharide from the mycelium of *Paecilomyces cicadae* TJJ1213. Food Res. Int..

[29] Tang C., Sun J., Zhou B., Jin C., Liu J., Gou Y., Chen H., Kan J., Qian C., Zhang N. (2018). Immunomodulatory effects of polysaccharides from purple sweet potato on lipopolysaccharide treated RAW 264.7 macrophages. J. Food Biochem..

[30] Wang X.M., Xu M.J., Xu D.L., Ma K., Zhang C.L., Wang G.X., Dong M.S., Li W. (2022). Structural and prebiotic activity analysis of the polysaccharide produced by *Lactobacillus helveticus* SNA12. Carbohydr. Polym..

[31] Cai H.W., Du F.N., Li L.Y., Li B.W., Li J.N., Shi H.H. (2019). A practical approach based on FT-IR spectroscopy for identification of semi-synthetic and natural celluloses in microplastic investigation. Sci. Total Environ..

[32] Liu Y.Y., Huang J.R., Zhu H.L., Liao P.Q., Chen X.M. (2023). Simultaneous Capture of CO_2_ Boosting Its Electroreduction in the Micropores of a Metal-organic Framework. Angew. Chem.-Int. Ed..

[33] Caliceti C., Calabria D., Roda A., Cicero A.F.G. (2017). Fructose Intake, Serum Uric Acid, and Cardiometabolic Disorders: A Critical Review. Nutrients.

[34] Jang C., Wada S., Yang S., Gosis B., Zeng X.F., Zhang Z.Y., Shen Y.H., Lee G., Arany Z., Rabinowitz J.D. (2020). The small intestine shields the liver from fructose-induced steatosis. Nat. Metab..

[35] Ren J. (2021). Bringing to fore the role of peptides, polyphenols, and polysaccharides in health: The research profile of Jiaoyan Ren. Food Front..

[36] Jang C., Hui S., Lu W., Cowan A.J., Morscher R.J., Lee G., Liu W., Tesz G.J., Birnbaum M.J., Rabinowitz J.D. (2018). The Small Intestine Converts Dietary Fructose into Glucose and Organic Acids. Cell Metab..

[37] Lambertz J., Weiskirchen S., Landert S., Weiskirchen R. (2017). Fructose: A Dietary Sugar in Crosstalk with Microbiota Contributing to the Development and Progression of Non-Alcoholic Liver Disease. Front. Immunol..

[38] Ziadi M., Bouzaiene T., M’Hir S., Zaafouri K., Mokhtar F., Hamdi M., Boisset-Helbert C. (2018). Evaluation of the Efficiency of Ethanol Precipitation and Ultrafiltration on the Purification and Characteristics of Exopolysaccharides Produced by Three Lactic Acid Bacteria. Biomed Res. Int..

[39] Angelin J., Kavitha M. (2020). Exopolysaccharides from probiotic bacteria and their health potential. Int. J. Biol. Macromol..

[40] Viesser J.A., Pereira G.V.D., Neto D.P.D., Rogez H., Goes-Neto A., Azevedo V., Brenig B., Aburjaile F., Soccol C.R. (2021). Co-culturing fructophilic lactic acid bacteria and yeast enhanced sugar metabolism and aroma formation during cocoa beans fermentation. Int. J. Food Microbiol..

[41] Senturk D.Z., Dertli E., Erten H., Simsek O. (2020). Structural and technological characterization of ropy exopolysaccharides produced by *Lactobacillus plantarum* strains isolated from Tarhana. Food Sci. Biotechnol..

[42] Zhang N., Zhang B., Chen X., Zhang Y., Wang Y., Lu S., Zhang H., Chen Y., Jiang H., Zhou H. (2024). Effects and mechanisms of Polygonati Rhizoma polysaccharide on potassium oxonate and hypoxanthine-induced hyperuricemia in mice. Int. J. Biol. Macromol..

[43] Xu Y.M., Cui Y.L., Wang X., Yue F.F., Shan Y.Y., Liu B.F., Zhou Y., Yi Y.L., Lu X. (2019). Purification, characterization and bioactivity of exopolysaccharides produced by *Lactobacillus plantarum* KX041. Int. J. Biol. Macromol..

[44] Xu Y.M., Cui Y.L., Yue F.F., Liu L.H., Shan Y.Y., Liu B.F., Zhou Y., Lü X. (2019). Exopolysaccharides produced by lactic acid bacteria and *Bifidobacteria*: Structures, physiochemical functions and applications in the food industry. Food Hydrocoll..

[45] Li S.Q., Shah N.P. (2015). Effects of *Pleurotus eryngii* polysaccharides on bacterial growth, texture properties, proteolytic capacity, and angiotensin-I-converting enzyme-inhibitory activities of fermented milk. J. Dairy Sci..

[46] Tang Y.J., Dong W., Wan K.Y., Zhang L.G., Li C., Zhang L.L., Liu N. (2015). Exopolysaccharide Produced by *Lactobacillus Plantarum* Induces Maturation of Dendritic Cells in BALB/c Mice. PLoS ONE.

[47] Kanmani P., Kumar R.S., Yuvaraj N., Paari K.A., Pattukumar V., Arul V. (2011). Production and purification of a novel exopolysaccharide from lactic acid bacterium *Streptococcus phocae* PI80 and its functional characteristics activity in vitro. Bioresour. Technol..

[48] Xu R.H., Shen Q., Wu R.Y., Li P.L. (2017). Structural analysis and mucosal immune regulation of exopolysaccharide fraction from *Bifidobacterium animalis* RH. Food Agric. Immunol..

[49] Wang J., Zhao X., Yang Y.W., Zhao A.M., Yang Z.N. (2015). Characterization and bioactivities of an exopolysaccharide produced by *Lactobacillus plantarum* YW32. Int. J. Biol. Macromol..

[50] Xu M., Xiao H., Zou X., Pan L., Song Q., Hou L., Zeng Y., Han Y., Zhou Z. (2025). Mechanisms of levan in ameliorating hyperuricemia: Insight into levan on serum metabolites, gut microbiota, and function in hyperuricemia rats. Carbohydr. Polym..

[51] Xu R.H., Shen Q.A., Ding X.L., Gao W.G., Li P.L. (2011). Chemical characterization and antioxidant activity of an exopolysaccharide fraction isolated from *Bifidobacterium animalis* RH. Eur. Food Res. Technol..

[52] Peng Y.F., Zhang L.N., Zeng F.B., Kennedy J.F. (2005). Structure and antitumor activities of the water-soluble polysaccharides from Ganoderma tsugae mycelium. Carbohydr. Polym..

[53] Wang K., Li W., Rui X., Chen X., Jiang M., Dong M. (2014). Characterization of a novel exopolysaccharide with antitumor activity from *Lactobacillus plantarum* 70810. Int. J. Biol. Macromol..

[54] Bomfim V.B., Pereira Lopes Neto J.H., Leite K.S., Vieira E.d.A., Iacomini M., Silva C.M., Olbrich dos Santos K.M., Cardarelli H.R. (2020). Partial characterization and antioxidant activity of exopolysaccharides produced by *Lactobacillus plantarum* CNPC003. Lwt-Food Sci. Technol..

[55] Hu S.-M., Zhou J.-M., Zhou Q.-Q., Li P., Xie Y.-Y., Zhou T., Gu Q. (2021). Purification, characterization and biological activities of exopolysaccharides from *Lactobacillus rhamnosus* ZFM231 isolated from milk. Lwt-Food Sci. Technol..

[56] Lu Y., Han S., Zhang S., Wang K., Lv L., McClements D.J., Xiao H., Berglund B., Yao M., Li L. (2022). The role of probiotic exopolysaccharides in adhesion to mucin in different gastrointestinal conditions. Curr. Res. Food Sci..

[57] Le M.T., Frye R.F., Rivard C.J., Cheng J., McFann K.K., Segal M.S., Johnson R.J., Johnson J.A. (2012). Effects of high-fructose corn syrup and sucrose on the pharmacokinetics of fructose and acute metabolic and hemodynamic responses in healthy subjects. Metab.-Clin. Exp..

[58] Tan J., Wan L., Chen X., Li X., Hao X., Li X., Li J., Ding H. (2019). Conjugated Linoleic Acid Ameliorates High Fructose-Induced Hyperuricemia and Renal Inflammation in Rats via NLRP3 Inflammasome and TLR4 Signaling Pathway. Mol. Nutr. Food Res..

[59] Muriel P., Lopez-Sanchez P., Ramos-Tovar E. (2021). Fructose and the Liver. Int. J. Mol. Sci..

[60] Choi J.W.J., Ford E.S., Gao X., Choi H.K. (2008). Sugar-sweetened soft drinks, diet soft drinks, and serum uric acid level: The third national health and nutrition examination survey. Arthritis Rheum.-Arthritis Care Res..

[61] Faller J., Fox I.H. (1982). Ethanol-induced hyperuricemia: Evidence for increased urate production by activation of adenine nucleotide turnover. N. Engl. J. Med..

[62] Meng W., Zhang J., Hou H., Yu L., Dong P. (2025). Exploring the structures and molecular mechanisms of bioactive compounds from marine foods for hyperuricemia prevention: A systematic review. Crit. Rev. Food Sci. Nutr..

[63] Zhu Y., Zhang R., Wei Y., Cai M., Ma Y., Gu R., Zhang H., Pan X. (2021). Rice peptide and collagen peptide prevented potassium oxonate-induced hyperuricemia and renal damage. Food Biosci..

[64] Chung S., Kim G.-H. (2021). Urate Transporters in the Kidney: What Clinicians Need to Know. Electrolyte Blood Press. E BP.

[65] Zhao J., Fu Y., Qiu H. (2025). Effect and mechanism of Plantaginis Semen polysaccharides on intestinal microecology in rats with hyperuricemia. Front. Microbiol..

[66] Softic S., Cohen D.E., Kahn C.R. (2016). Role of Dietary Fructose and Hepatic De Novo Lipogenesis in Fatty Liver Disease. Dig. Dis. Sci..

[67] Jegatheesan P., De Bandt J.P. (2017). Fructose and NAFLD: The Multifaceted Aspects of Fructose Metabolism. Nutrients.

[68] Livesey G. (2010). Fructose, Obesity, and Related Epidemiology. Crit. Rev. Food Sci. Nutr..

[69] Joosten L.A.B., Crisan T.O., Bjornstad P., Johnson R.J. (2020). Asymptomatic hyperuricaemia: A silent activator of the innate immune system. Nat. Rev. Rheumatol..

[70] Wang Z., Zhang Z., Lu C., Zhou J., Wang Z., Han J., Su X. (2022). Effects of *Sporisorium reiliana* polysaccharides and Phoenix dactylifera monosaccharides on the gut microbiota and serum metabolism in mice with fructose-induced hyperuricemia. Arch. Microbiol..

[71] Wang L.P., Ji T.T., Yuan Y., Fu H.Y., Wang Y., Tian S.B., Hu J., Wang L., Wang Z. (2022). High-fructose corn syrup promotes proinflammatory Macrophage activation via ROS-mediated NF-Kappa B signaling and exacerbates colitis in mice. Int. Immunopharmacol..

[72] Guo Z., Zhang J., Wang Z., Ang K.Y., Huang S., Hou Q., Su X., Qiao J., Zheng Y., Wang L. (2016). Intestinal Microbiota Distinguish Gout Patients from Healthy Humans. Sci. Rep..

[73] Yu Y., Liu Q., Li H., Wen C., He Z. (2018). Alterations of the Gut Microbiome Associated With the Treatment o f Hyperuricaemia in Male Rats. Front. Microbiol..

[74] Chu Y., Sun S., Huang Y., Gao Q., Xie X., Wang P., Li J., Liang L., He X., Jiang Y. (2021). Metagenomic analysis revealed the potential role of gut microbiome in gout. npj Biofilms Microbiomes.

[75] Liu Z.B., Chen Z.C., Guo H.W., He D.P., Zhao H.R., Wang Z.Y., Zhang W., Liao L., Zhang C., Ni L. (2016). The modulatory effect of infusions of green tea, oolong tea, and black tea on gut microbiota in high-fat-induced obese mice. Food Funct..

[76] Kong C., Gao R.Y., Yan X.B., Huang L.S., Qin H.L. (2019). Probiotics improve gut microbiota dysbiosis in obese mice fed a high fat or high-sucrose diet. Nutrition.

