# Supplementary material for: Exopolysaccharides from Lacticaseibacillus rhamnosus Fmb14 Ameliorate Fructose-Induced Hyperuricemia and Fatty Liver via Gut Modulation
Source: Foods. 2026 Jan 23;15(3):409. doi: 10.3390/foods15030409 (PMC12896772; doi:10.3390/foods15030409)
Supplement: Supplementary file 1 [file foods-15-00409-s001.zip › foods-4097787-supplementary.pdf]

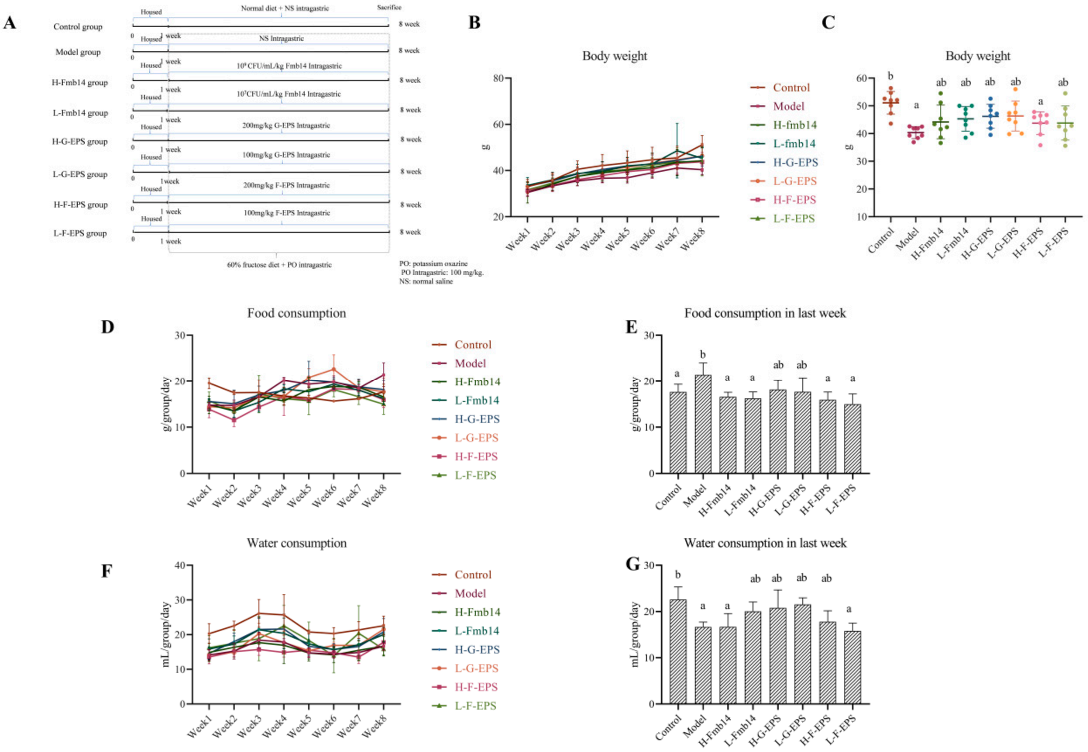

Figure S1. EPS intervention altered the conditions of high purine consumption in mice. A) Experimental design for the treatment of hyperuricemic mice. B-C) Body weight fluctuations of the mice throughout the 8-week period. D-E) Food consumption fluctuations in mice throughout the 8-week period. F-G, Water consumption fluctuations in the mice throughout the 8-week period.

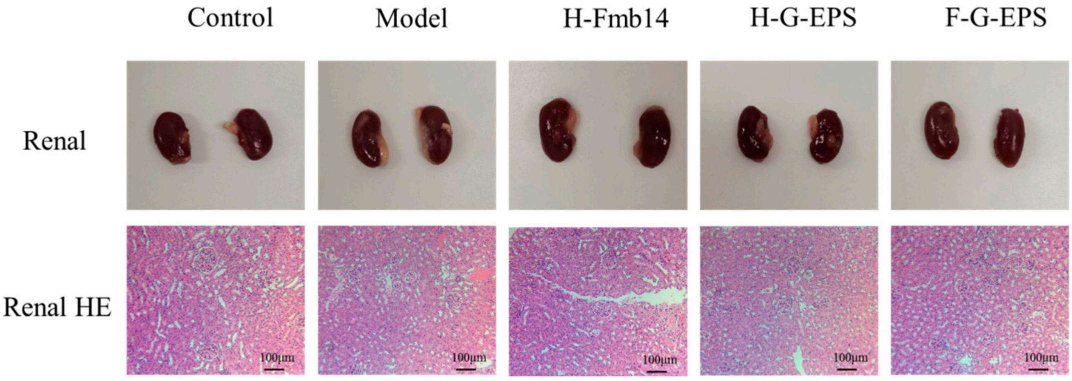

Figure S2. The effect of *L. rhamnosus* Fmb14 and EPS treatment on renal in hyperuricemic mice.

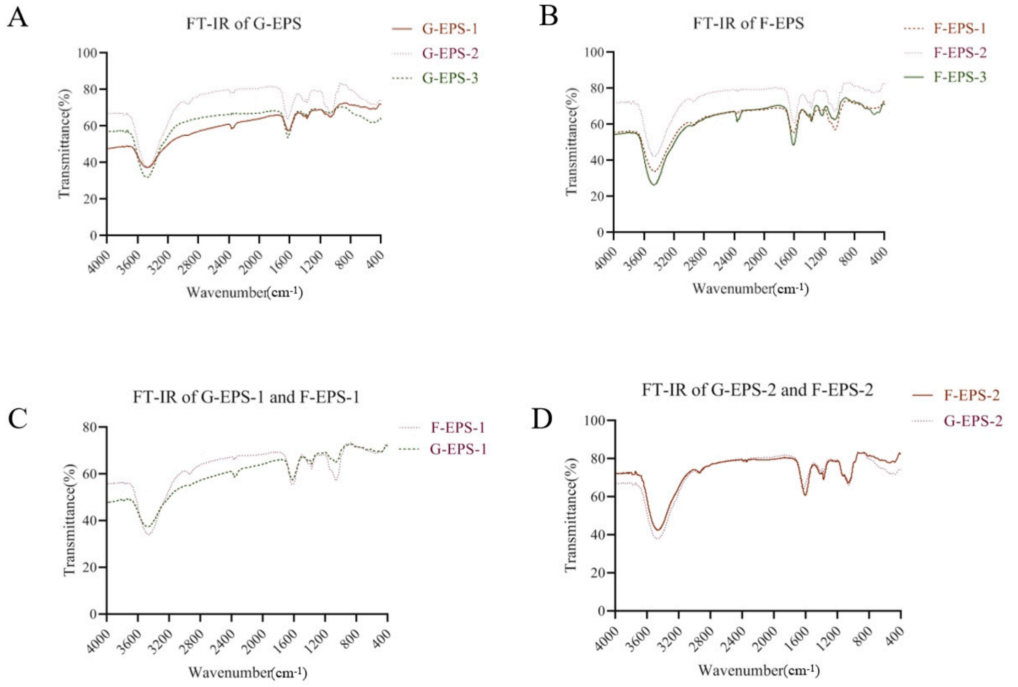

Figure S3. FT-IR spectra of G-EPSs and F-EPSs from *L. rhamnosus* Fmb14
